# The Correlation Between Triglyceride–Glucose–Body Mass Index, and the Risk of Silent Myocardial Infarction: Construction of a Predictive Model

**DOI:** 10.31083/RCM36608

**Published:** 2025-07-23

**Authors:** Rong Feng, Jiahui Lu, Honggen Cui, Yaqin Li

**Affiliations:** ^1^Department of Cardiology, Affiliated Hospital of Hebei University, 071030 Baoding, Hebei, China; ^2^Department of Endocrinology, Affiliated Hospital of Hebei University, 071030 Baoding, Hebei, China

**Keywords:** silent myocardial infarction, clinically manifested myocardial infarction, triglyceride glucose-body mass index, prediction model

## Abstract

**Background:**

The incidence of silent myocardial infarction (SMI) is increasing. Meanwhile, due to the atypical clinical symptoms and signs associated with SMI, the prognosis for patients is often poor.

**Methods:**

This prediction model used the least absolute shrinkage and selection operator (LASSO) and multivariate logistic regression analyses to screen variables. Predictive accuracy was assessed using the area under the receiver operating characteristic (ROC) curve (AUC). The clinical decision curve analysis (DCA), alongside the calibration curve and clinical impact curve (CIC) analyses, were used to assess model validity.

**Results:**

This study included 174 patients, 64 (36.8%) of whom experienced SMI; logistic regression analysis identified six variables: gender, age, high-density lipoprotein cholesterol (HDL-C), apolipoprotein B/apolipoprotein A1 (ApoB/A1), uric acid (UA), and triglyceride glucose–body mass index (TyG–BMI). The results identified the TyG–BMI as a predictor of SMI (odds ratios (OR) = 1.02, 95% CI: 1.01–1.03;* p *= 0.003). The ROC curve of the model demonstrated an AUC of 0.772 (95% CI: 0.699–0.844), which increased to 0.774 (95% CI: 0.707–0.841) following a bootstrap analysis with 1000 repetitions. The calibration curve of the model was in high agreement with the ideal curve. The DCA demonstrated that the prediction probability threshold of the model ranged from 12% to 83%, where the patient achieved a significant net clinical benefit. The CIC showed that the model effectively identified high-risk SMI patients when the threshold probability exceeded 0.7.

**Conclusions:**

The TyG–BMI is an independent predictor of SMI. A prediction model based on the TyG–BMI showed good predictive ability for SMI.

## 1. Introduction

Myocardial infarction (MI) is a common and serious disease characterized by poor prognosis and high mortality. If patients with clinically manifested myocardial infarction (CMMI) can receive timely and effective treatment, fewer serious cardiovascular events occur [1]. However, due to changes in diet and lifestyle, the incidence of silent myocardial infarction (SMI) in patients with MI has continued to rise [2]. Patients with SMI are often discovered during physical examinations or cardiac function testing for other conditions. Due to they have few or no apparent symptoms, they are not often identified in time to receive prompt treatment [3]. This may result in cardiovascular complications which adversely affect long-term prognosis [4]. Cheng *et al*. [5] indicated a significant association between SMI and long-term risk of cardiovascular death, with a hazard ratio (HR) of 5.20 (95% CI: 3.81–7.10).

Several models have been developed to show that the TyG index [6], fasting glucose [7] and gender [8] are independent risk factors for the development of SMI. However, these studies have only validated the individual effects of these variables and no further investigation of their collective role in assessing the risk of developing SMI has occurred until now.

The triglyceride glucose-body mass index (TyG-BMI) provides a more comprehensive risk assessment as it reflects both insulin resistance (IR) and the impact of obesity on cardiovascular health [9, 10]. Additionally, TyG-BMI is a better predictor of coronary heart disease than other indices. A study evaluating the predictive ability of several indicators of insulin resistance for new-onset coronary heart disease found that the TyG-BMI index demonstrated the highest level of predictive accuracy, with an area under the receiver operating characteristic curve (AUC) of 0.609 [11, 12]. It surpassed the TyG index (AUC = 0.595), TyG-Waist Circumference (TyG-WC, AUC = 0.606), and TyG-Waist-to-Height Ratio (TyG-WHtR, AUC = 0.609). Further, TyG-BMI is calculated from triglycerides, blood glucose and BMI. Clinical data are readily available and more convenient to use.

It is hypothesized here that the TyG-BMI index could be used as an independent predictor of SMI, the aim being to develop a predictive model that improves clinical diagnosis.

## 2. Materials and Methods

### 2.1 Study Population

**Fig. 1.**
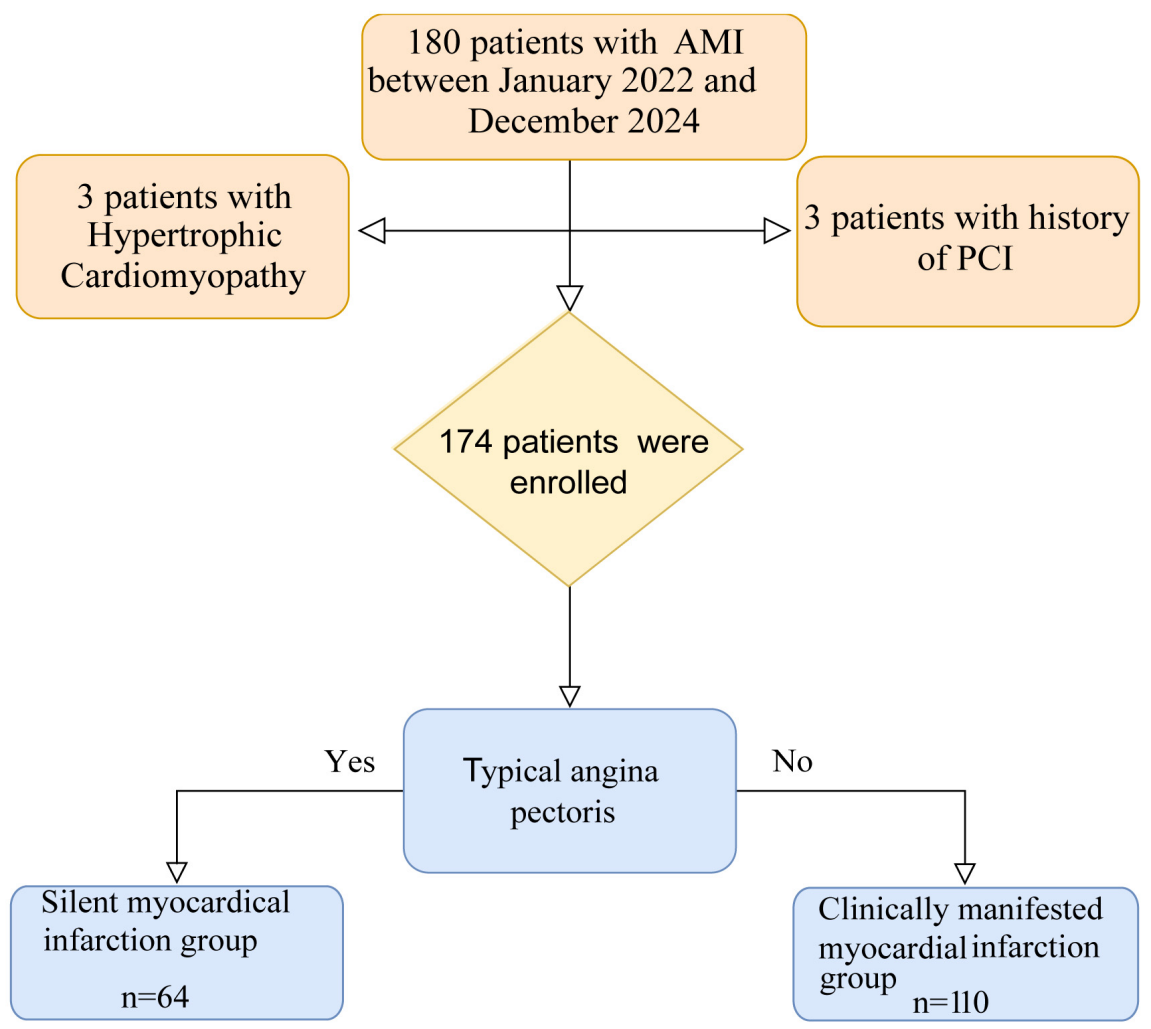
**Flow diagram of the study**. AMI, acute myocardial infarction; PCI, percutaneous coronary intervention.

This retrospective study included myocardial infarction patients who received treatment in the Cardiology Department of Hebei University Affiliated Hospital from January 2022 to December 2024. Inclusion Criteria. (1) Age ≥18 years. (2) The definition of SMI from the Fourth Universal Definition of Myocardial Infarction (2018) [13]. (3) Patients exhibiting typical symptoms of angina pectoris, dynamic changes in the ST-T segments of the electrocardiogram and elevated levels of myocardial enzymes were included in the CMMI group. Exclusion Criteria: (1) Patients with a documented medical history of percutaneous coronary intervention, coronary artery bypass grafting, hypertrophic cardiomyopathy (HCM), and stroke. (2) Patients suffering from cachexia, including those afflicted with hematological disorders or tumors. A flow chart of the screening of the study population is given in Fig. 1.

### 2.2 Sample Size Calculation

The per event variable (EPV) method was used to calculate the sample size [14]. According to the protocol, it was planned to include six predictor variables and set the per event variable to 10 so that 60 patients with SMI would be required to ensure reliable predictive modeling. The prevalence of SMI among MI patients was 36.8% in this study. Allowing for a potential 5% data loss, a total of 174 patients were included. The final sample size was calculated as follows:

$$S\,a\,m\,p\,l\,e\,S\,i\,z\,e\,=\frac{N\,u\,m\,b\,e\,r\,o\,f\,V\,a\,r\,i\,a\,b\,l\,e\,s\times E\,P\,V}{1-I\,n\,c\,i\,d\,e\,n\,c\,e\,R\,a\,t\,e}=\frac{6\times 10}{1-0.368}\times \left(1+5\%\right)=171$$

### 2.3 Ethical Approval

This study was approved and supported by the Ethics Review Committee of Hebei University Affiliated Hospital(Protocol No. HDFY-LL-2022-058). All patients provided informed consent for the procedure as well as the subsequent data collection and analysis for research.

### 2.4 Disease Definition

#### 2.4.1 CMMI

The patient complained of severe retrosternal chest pain lasting more than 30 minutes and ineffective treatment with nitrates. The electrocardiogram (ECG) showed ST-segment depression (≥0.1 mV) with T-wave inversion in two adjacent leads, which changed over time. Myocardial markers were abnormal and coronary angiography showed complete arterial occlusion. During the non-acute period, patient electrocardiographic performance was the same as that of patients with SMI [15].

#### 2.4.2 SMI

All patients had no chest pain or only mild symptoms of chest tightness. However, the ECG showed ST-segment elevation (≥0.1 mV) with T-wave inversion in two adjacent leads. The ECG showed dynamic ST-T changes. Myocardial markers were abnormal, and coronary angiography showed complete arterial occlusion. In the non-acute phase, pathological Q waves may be present in two adjacent leads on the ECG [13].

#### 2.4.3 TyG-BMI Index 

Triglyceride (TG), fasting plasma glucose (FPG), height and weight were collected on the day of admission, and TyG-BMI was calculated according to the following formula [16]:

TyG-BMI = ln[TG × 88.545 (mmol/L) × FPG × 18.02 (mmol/L)/2] × BMI (kg/m^2^)

#### 2.4.4 Gensini Score 

The severity of coronary artery disease was quantitatively assessed using the Gencini score system. The score consisted of two components. Coronary artery stenosis was scored as, 1: stenosis ≤25%, 2: stenosis 26%–50%, 4: stenosis 51%–75%, 8: stenosis 76%–90%, 16: stenosis 91%–99%, and 32: stenosis 100%. Scores for different lesion locations in the coronary arteries included: left aorta, 5 points; anterior descending artery, 2.5 points proximal, 1.5 points mid, 1.0 point distal to first diagonal, 0.5 point second diagonal; circumflex artery, 2 points, circumflex artery, 2.5 points proximal, 1.5 points middle, 1.0 point distal to first diagonal, 0.5 point second diagonal; circumflex artery, 2.5 points proximal, 1.5 points middle, 1.0 point distal to obtuse marginal branch; right coronary artery, 1.0 point proximal, 1.0 point middle distal, 1.0 point distal and 1.0 point posterior to descending and 0.5 point posterior to left ventricular. Finally, the score determined by the site of the lesion was multiplied by the coefficient. If there were multiple lesions, the total score was calculated [17].

### 2.5 Collection of Clinical Data

General patient information was collected through the hospital’s electronic medical record system. These included age, gender, BMI, and medical history such as hypertension, diabetes, smoking, and drinking. Smoking history was defined as six months or longer of continuous or cumulative smoking. Drinking was assessed based on whether the patient had consumed alcoholic beverages in the past year, six months, or one month. Former smokers or drinkers are defined as those who have quit for less than one year [18]. Hypertension is described as having a history of hypertension or having two or more readings during admission with a systolic blood pressure ≥140 mmHg or diastolic blood pressure ≥90 mmHg [19]. Diabetes is defined as having a history of diabetes or having FPG ≥7.0 mmol/L, an oral glucose tolerance test two hours blood glucose ≥11.1 mmol/L, or random blood glucose ≥11.1 mmol/L, along with symptoms of hyperglycemia [20]. Laboratory test results from the day of admission were collected, including neutrophil percentage (N%), lymphocyte percentage (L%), monocyte percentage (M%), total cholesterol (TC), TG, high-density lipoprotein C (HDL-C), low-density lipoprotein C (LDL-C), apolipoprotein B/apolipoprotein A1 (ApoB/A1), aspartate aminotransferase (AST), alanine aminotransferase (ALT), FPG, serum creatinine (Scr), and uric acid (UA). Echocardiographic parameters, including left ventricular end-diastolic diameter (LVEDD) and ejection fraction (LVEF). Information about medication use after discharge was collected, including antiplatelet agents, statins, β-blockers, angiotensin-converting enzyme inhibitor (ACEI), angiotensin receptor blocker (ARB) and calcium channel blockers.

### 2.6 Statistical Methods

SPSS (version 27.0, IBM Corp., Chicago, IL, USA) and R software (version 4.4.1, R Foundation for Statistical Computing, Vienna, Austria) were used for statistical data analysis. Data were preprocessed using the mice package in R for multiple imputations. Variables or individual data with a missing rate >20% were excluded, while those with a missing rate ≤20% were imputed. Subsequently, the Shapiro–Wilk test was used to assess the normality of continuous data. Normally, distributed continuous data are presented as mean ± SD. Non-normal data are summarized as median (25th, 75th percentiles). Categorical variables are expressed as counts and percentages. The independent samples *t*-test or Mann-Whitney U test was employed to compare continuous variables between groups. The chi-square test was employed for categorical variables. The least absolute shrinkage and selection operator (LASSO) and multivariable logistic regression were employed to select associated variables. A nomogram model was developed and its discriminatory power evaluated, calibration curves for assessing calibration were plotted, the C-index calculated and the Hosmer-Lemeshow test was used to quantify its performance. Bootstrap validation (×1000 resamples) was performed and the adjusted C-index was calculated. The calibration curves and clinical decision curve analysis (DCA) were used to quantify the net benefit at different probability thresholds and to assess the model’s clinical utility. The clinical impact curves (CIC) was used to evaluate the model’s further applicability. A *p*-value < 0.05 was considered statistically significant.

## 3. Results

### 3.1 General Baseline Characteristics

**Fig. 2.**
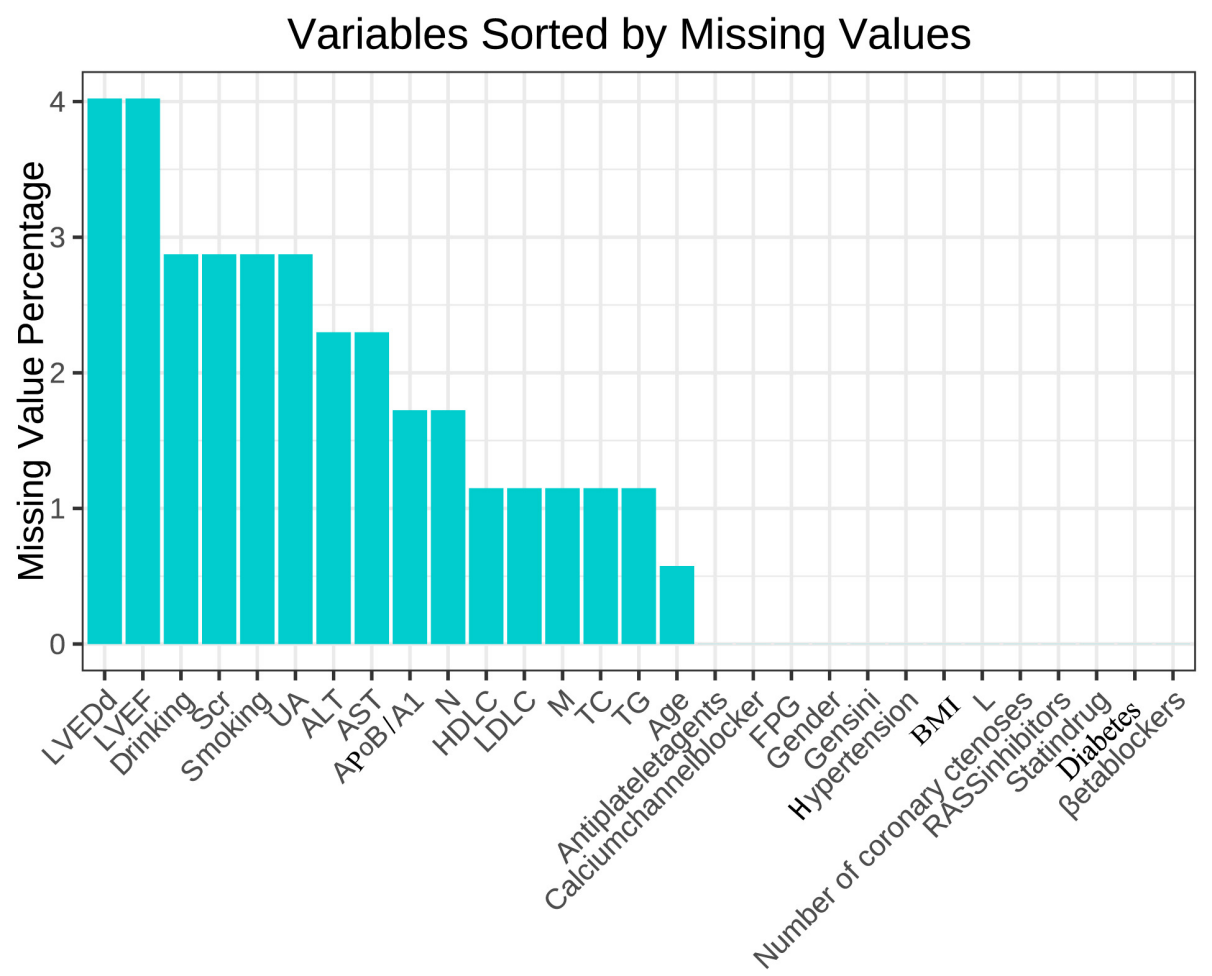
**Missing values of variables**.

There were 17 missing variables (<20%), as detailed in Fig. 2. Table 1 listed the baseline clinical characteristics of patients, divided into the SMI and the clinically manifested myocardial infarction (CCMI) groups. The average age was 61.68 ± 10.4 years, with 106 males (60.9%) and 68 females (39.1%). A total of 64 patients (36.8%) experienced SMI. The SMI group had higher levels of male patients, hypertension history, smoking history, BMI, TG, ApoB/A1, UA, LVEDD, Gensini score, FPG and TyG-BMI when compared to the CCMI group. In contrast, the SMI group had lower levels of HDL-C (*p* < 0.05). Age, drinking, diabetes, N%, L%, M%, TC, LDL-C, AST, ALT, Scr, LEVF, number of coronary artery stenoses, and medication history were not significant in in either group.

**Table 1.** **Baseline characteristics of participants**.

Variables		Groups		*p-value*
		SMI Group	CMMI Group	
		(n = 64)	(n = 110)	
Age (years, $\bar{\chi }$ ± s)		62.95 ± 11.67	60.95 ± 9.72	0.260
Gender (male) [n (%)]		47 (73.44)	59 (53.64)	0.010
Hypertension [n (%)]		45 (70.31)	58 (52.73)	0.023
Diabetes [n (%)]		18 (28.13)	25 (22.73)	0.426
BMI (kg/m^2^, $\bar{\chi }$ ± s)		26.35 ± 2.73	25.25 ± 2.77	0.012
Smoking [n (%)]		28 (43.75)	30 (27.27)	0.026
Drinking [n (% )]		17 (26.56)	21 (19.09)	0.250
N% [%, $\bar{\chi }$ ± s]		63.00 ± 9.56	62.44 ± 8.52	0.691
L% [%, M (Q1, Q3)]		27.85 (21.50, 33.18)	27.80 (22.76, 32.50)	0.933
M% [%, M (Q1, Q3)]		6.95 (5.33, 8.50)	6.80 (5.90, 8.13)	0.521
TC [mmol/L, M (Q1, Q3)]		4.19 (3.58, 4.59)	3.93 (3.20, 4.81)	0.313
TG [mmol/L, M (Q1, Q3)]		1.84 (1.1, 2.6)	1.44 (1.07, 2.11)	0.033
HDL‑C [mmol/L, M (Q1, Q3)]		0.93 (0.77, 1.07)	0.94 (0.87, 1.15)	0.032
LDL‑C [mmol/L, M (Q1, Q3)]		2.56 (2.08, 3.12)	2.36 (1.77, 3.19)	0.458
ApoB/A1 [M (Q1, Q3)]		1.87 (1.43, 2.40)	1.64 (1.24, 2.23)	0.024
AST [U/L, M (Q1, Q3)]		19.50 (16.00, 23.18)	20.0 (16.00, 25.00)	0.659
ALT [U/L, M (Q1, Q3)]		18.00 (14.00, 24.00)	20.0 (14.00, 28.25)	0.535
Scr [µmol/L, M (Q1, Q3)]		69.50 (61.50, 76.00)	67.50 (55.00, 79.25)	0.455
UA [µmol/L, M (Q1, Q3)]		362.50 (319.15, 408.00)	336.00 (290.25, 390.00)	0.013
LVEDD [mm, M (Q1, Q3)]		48.79 (46.25, 49.00)	47.20 (46.00, 48.00)	<0.001
LVEF [%, M (Q1, Q3)]		62.72 (60.18, 66.00)	62.19 (62.14, 65.00)	0.662
FPG [mmol/L, M (Q1, Q3)]		7.6 (6.00, 8.78)	6.50 (5.38, 8.20)	0.005
Gensini score [points, M (Q1, Q3)]		48.00 (42.75, 49.00)	40 (25.00, 50.00)	0.006
TyG-BMI ($\bar{\chi }$ ± s)		245.59 ± 33.74	227.67 ± 31.93	<0.001
Number of coronary stenoses [n, (%)]				0.508
	<2	14 (21.90)	29 (26.40)	
	≥2	50 (78.10)	81 (73.60)	
Drug Class [n, (%)]				
	Antiplatelet Agents	64 (100.00)	110 (100.00)	1.000
	Statins	64 (100.00)	110 (100.00)	1.000
	β-blockers	28 (43.75)	39 (35.45)	0.278
	ACEI/ARB	3 (4.69)	3 (2.73)	0.801
	Calcium Channel Blockers	26 (40.63)	33 (30.00)	0.153

BMI, body mass index; N%, neutrophil percentage; L%, lymphocyte percentage; M%, monocyte percentage; TC, total cholesterol; TG, triglycerides; HDL-C, high-density lipoprotein C; LDL-C, low-density lipoprotein C; ApoB/A1, apolipoprotein B/apolipoproteinA1; AST, aspartate aminotransferase; ALT, alanine aminotransferase; Scr, serum creatinine; UA, uric acid; LVEDD, left ventricular end-diastolic diameter; LVEF, left ventricular ejection fraction; FPG, fasting plasma glucose; TyG-BMI, triglyceride-glucose-body mass index; ACEI, angiotensin-converting enzyme inhibitor; ARB, angiotensin receptor blocker; SMI, silent myocardial infarction; CMMI, clinically manifested myocardial infarction.

### 3.2 Correlation Heatmap of the Predictor Variables 

**Fig. 3.**
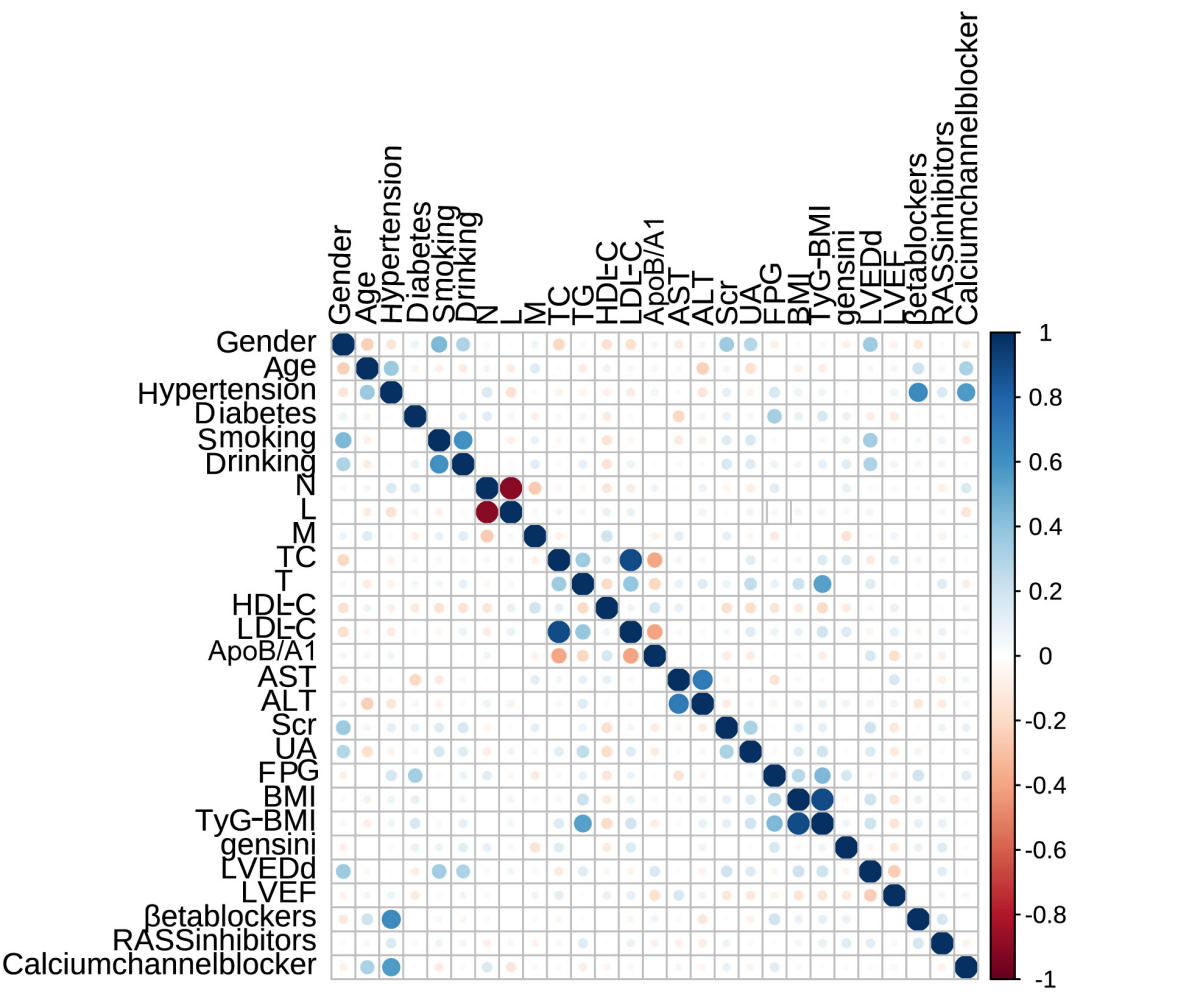
**Correlation matrix of variables**.

Fig. 3 showed the correlation matrix for all variables. Light-colored cells indicate weak interactions. Dark blue and red areas represented stronger positive and negative correlations, respectively. The figure showed that FPG was significantly positively correlated with BMI, TG and TyG-BMI. Additionally, results also suggest a weak correlation between UA, TyG-BMI and gender, which may influence the direct association of these variables with outcome events.

### 3.3 Screening of Risk Factors

**Fig. 4.**
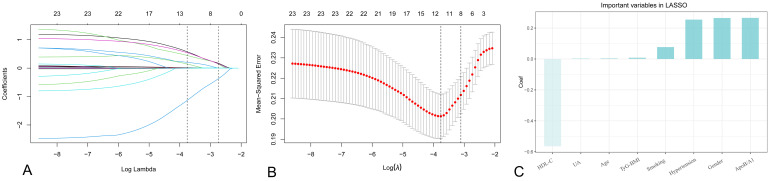
**Results of the LASSO logistic regression**. (A) The LASSO regression analysis selected from 23 variables those that were highly associated with the onset of SMI. (B) Ten-fold cross-validation. (C) The ranking of variable importance in the LASSO logistic regression. LASSO, least absolute shrinkage and selection operator.

TyG-BMI was calculated from TG, FPG and BMI. The confusion matrix shows a significant correlation between these variables. To avoid multicollinearity, these variables were removed from Table 1. A LASSO regression analysis with 10-fold cross-validation identified the optimal lambda (λ) value as 0.04463813. Eight risk factors were selected: gender, age, hypertension, smoking, HDL-C, Apo B/A1, UA and TyG-BMI (Fig. 4).

### 3.4 Selection of Variables and Model Establishment

**Fig. 5.**
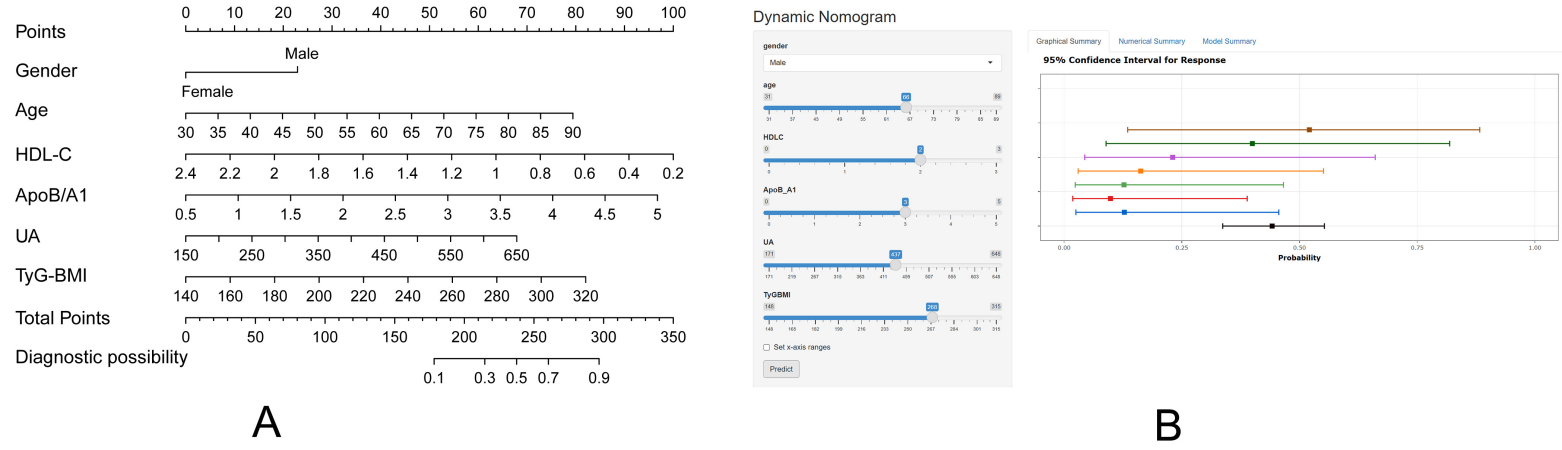
**Nomogram of the prediction model**. (A) The nomogram for predicting the risk of SMI includes parameters such as HDL-C, Age, Gender, ApoB/A1, UA, and TyG-BMI. (B) An online interactive graph can be accessed at https://myname.shinyapps.io/dynnomapp/.

The eight risk variables selected by LASSO regression as independent variables and the occurrence of SMI as the dependent variable were used to perform multivariable logistic regression via backward stepwise regression. Results showed that Gender, Age, ApoB/A1, UA, and TyG-BMI were independent risk factors for SMI and HDL-C was a protective factor (Table 2, *p* < 0.05). A nomogram model was developed (see https://myname.shinyapps.io/dynnomapp/, Fig. 5B) to predict the risk of SMI (Fig. 5A). The prediction formula of the model is Logit(P) = –9.762–1.687 × HDL-C + 0.049 × Age + 0.851 × Gender + 0.798 × ApoB/A1 + 0.005 × UA + 0.017 × TyG-BMI.

**Table 2.** **Multivariate logistic regression analysis of SMI**.

Variables	Multivariate logistic regression analysis					
	β	SE	Wald	OR values	95% CI	*p* values
Gender (Male)	0.851	0.399	4.551	2.34	(1.07, 5.12)	0.033
Age	0.049	0.019	6.674	1.05	(1.01, 1.09)	0.010
HDL‑C	–1.687	0.832	4.113	0.19	(0.04, 0.95)	0.043
ApoB/A1	0.798	0.277	8.276	2.22	(1.29, 3.83)	0.004
UA	0.005	0.002	4.460	1.01	(1.00, 1.01)	0.035
TyG-BMI	0.017	0.006	8.674	1.02	(1.01, 1.03)	0.003
Constant	–9.762	2.567	14.459	0.000	–	<0.001

SE, standard error.

### 3.5 Calibration and Discrimination Ability of the Model

**Fig. 6.**
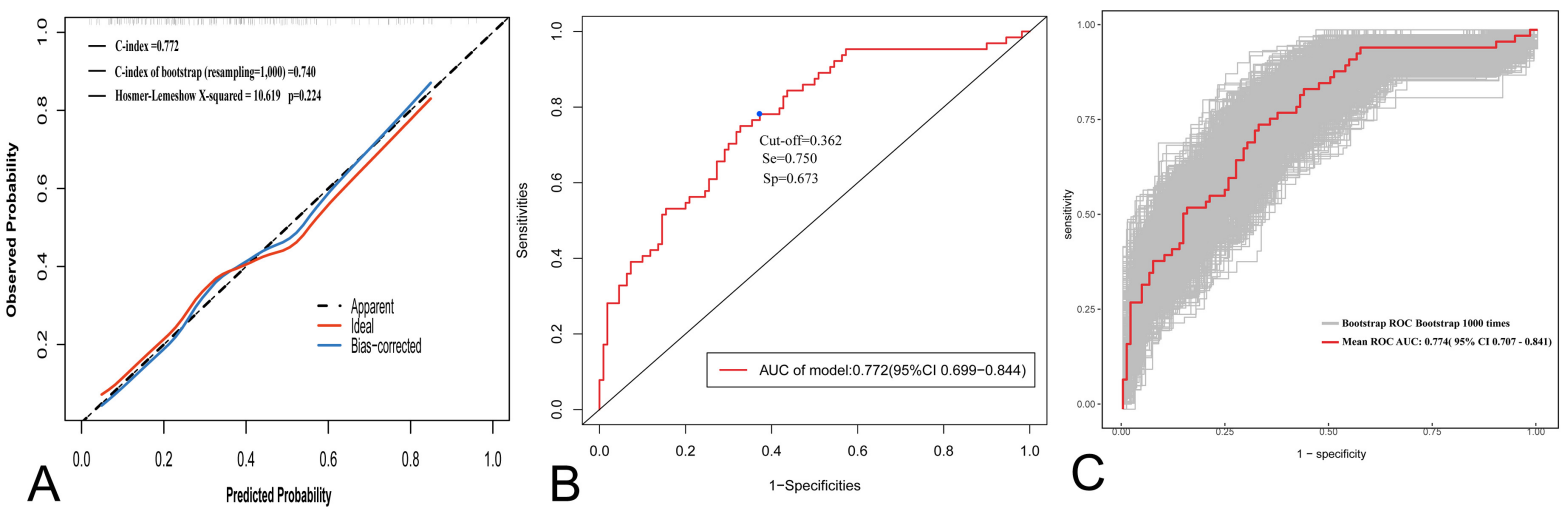
**Discriminative power and accuracy of nomogram of the prediction model**. (A) Calibration curve of the SMI nomogram prediction model. (B) The ROC curve of the SMI prediction model. (C) The ROC curve of the SMI prediction model with bootstrap (resampling = 1000). se, sensitivity; sp, specificity.

The calibration curve showed that the predictions of the model were very similar to the ideal diagonal during 1000 iterations, which verified the good reliability (Fig. 6A). The receiver operating characteristic (ROC) curve analysis showed a pre-sampling AUC of 0.772 (95% CI: 0.699–0.844), with an optimal cutoff value of 0.362 (sensitivity: 0.750, specificity: 0.673) (Fig. 6B). The post-sampling AUC was 0.774 (95% CI: 0.707–0.841) (Fig. 6C), supporting the robust discriminatory ability of the model. The result of the Hosmer-Lemeshow test was X^2^ = 10.619 (*p* = 0.2242), indicating no significant deviation between predicted and actual risk. The C-index was 0.772, and the bootstrap-validated C-index (1000 resamples) was 0.740. The results of the study confirmed that the predictive model to have good fit, discriminative power and credibility and can be used to is suited to prediction of SMI risk.

### 3.6 Clinical Application of the Risk Model

**Fig. 7.**
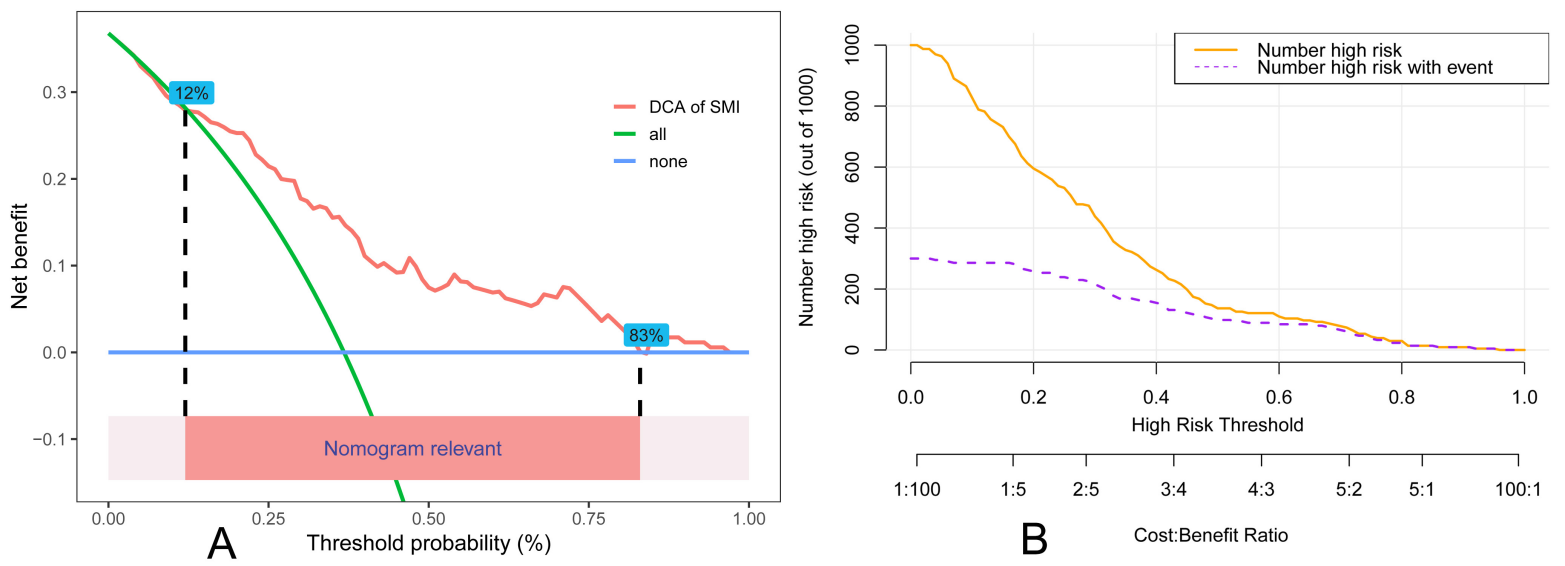
**The DCA and CIC of the SMI risk model**. (A) Decision curve analysis. (B) The clinical impact curve analysis. DCA, decision curve analysis; CIC, clinical impact curves.

A DCA and an assessment of CIC was performed for the SMI risk model. Results of the DCA showed that the model had a higher net benefit when the risk threshold was set between 12% and 83%, indicating that it provides substantial evidence for clinical decision-making (Fig. 7A). In the analysis of the CIC, it was observed that the predictive model was able to accurately identify individuals at high risk for SMI at a risk threshold of 0.7 (Fig. 7B). The results of the DCA and CIC showed that the false-positive rate was within an acceptable range, demonstrating the potential of the model to reduce the risk of unnecessary treatment. The critical value of the model in clinical practice helps clinicians make risk assessment decisions and improves the effectiveness of patient management.

## 4. Discussion

The number of silent myocardial infarctions in patients with myocardial infarction is increasing and contributes significantly to the poor prognosis of these patients. In the current study, 36.8% of patients with MI had SMI, a rate similar to previous studies.

This study shows that six factors, including TyG-BMI, gender, age, HDL-C, ApoB/A1 and UA, are associated with SMI. The model based on the TyG-BMI index had high accuracy (AUC = 0.772, 95% CI: 0.699–0.844) for the occurrence of SMI and the model had good predictive efficacy (C-index = 0.772). The model also had a significant net clinical benefit over a threshold range of 12%–83%. When predicting the occurrence of SMI in 1000 MI patients, the model could accurately identify high-risk individuals with a risk threshold greater than 0.7, strongly agreeing with the actual occurrence of SMI.

SMI leads to a poor prognosis due to its lack of apparent symptoms, with its pathological mechanisms may including: (1) With age, a person’s pain threshold increases, leading to decreased pain sensitivity [21]. (2) Prolonged exposure to risk factors such as hypertension and hyperlipidemia affects the function of autonomic and sensory nerves, leading to hyperalgesia or even loss of sensation [22]. (3) The inflammatory response is involved in the entire process of atherosclerosis. Studies have shown that Th2-type lymphocytes can make patients less sensitive to pain by promoting increased release of endorphins and anti-inflammatory cytokines such as interleukin 4 and interleukin 10 [23].

The TyG-BMI index is an important indicator for assessing the risk of metabolic syndrome and cardiovascular disease. It helps to identify high-risk groups at an early stage and to reduce the risk of developing SMI. Although there are no studies directly linking TyG-BMI to the onset and progression of SMI, a related study of cardiovascular disease prognosis involving 370,390 participants (HR = 1.47, 95% CI: 1.39–1.56) suggests that TyG-BMI is an independent risk factor for myocardial infarction [24]. The TyG-BMI is an important risk factor for myocardial infarction, possibly by modulating insulin resistance and lipid metabolism. First, the role of insulin resistance on MI is multifaceted. (1) Insulin resistance induces chronic inflammation in the body by promoting the release of inflammatory factors such as TNF-α and interleukin 6. This damages to the vascular endothelium and causes the formation of atherosclerotic plaques, increasing the risk of atherosclerosis [25]. (2) By interfering with endothelial cell function and thereby reducing nitric oxide (NO) production, it causes vasoconstriction and exacerbates myocardial ischemia [26]. (3) Insulin resistance triggers oxidative stress (OS) and increases the production of reactive oxygen species (ROS), further damaging cardiomyocytes and vascular endothelial cells [27]. Then, BMI may in some sense reflect the amount of fat in the body. In obese people, excessive fat accumulation can inhibit the activity of hepatic LDL receptors, which in turn reduces their clearance, and LDL accelerates atherosclerosis by contributing to plaque formation through deposition in the vascular endothelium. Additionally, obesity promotes the secretion of inflammatory factors and destabilizes plaques, increasing the risk of myocardial infarction [28].

Additionally, gender is closely related to the occurrence of SMI. Hummel *et al*. [29] reported that the incidence of SMI was 0.3% in women and 0.6% in men, with a prevalence rate that was 50% lower in women than in men (OR = 0.50, 95% CI: 0.33–0.75, *p* < 0.01). This finding is consistent with our results. This difference may be related to the anti-atherosclerotic effects of estrogen in women, such as anti-inflammation and improvement of vascular endothelial function. In addition, men are more likely to underestimate atypical symptoms, which may delay the recognition of MI.

Both HDL-C and the Apo B/A1 ratio are related to lipid metabolism. HDL-C transmits cholesterol from peripheral tissues to the liver, inhibiting the oxidation of LDL, reducing the release of inflammatory factors, and promoting the release of NO, thus inhibiting atherosclerotic plaque formation. The ApoB/A1 ratio reflects the balance between pro-atherogenic and anti-atherogenic lipoproteins. A higher ApoB/A1 ratio indicates elevated cardiovascular risk, while a lower ratio suggests stronger protection. Hua R *et al*. [30], demonstrated that ApoB/A1 is a risk factor for multivessel coronary disease (OR = 2.768, 95% CI: 1.868–4.103, *p* < 0.001). Hyperuricemia may increase myocardial infarction risk by promoting atherosclerosis (vascular endothelial damage, inflammation, oxidative stress) and metabolic disorders (insulin resistance) [31]. A cross-sectional study and Mendelian randomization analysis has further shown higher UA levels significantly increased the risk of MI. The study included 23,080 participants in a cross-sectional analysis (OR = 2.843, 95% CI: 1.296–6.237, *p* = 0.010). Mendelian randomization analysis further shown that genetically higher UA levels made the risk of MI significant (OR = 1.333, 95% CI: 1.079–1.647, *p* = 0.008) [32].

Li and Yu [33] developed a prediction model for the risk of SMI using five variables: age, sex, diabetes, history of MI, and history of hyperlipidemia. The model showed good predictive ability (AUC = 0.74, 95% CI: 0.67–0.82, *p* < 0.001). In this study, the patient’s glucose, lipid and obesity were combined to include TyG-BMI as an additional variable in the model. The current model has a higher AUC of 0.772 and better predictive ability for high-risk groups, which compensates for the poor assessment of metabolic abnormalities in patients with SMI by the former model [33]. In addition, the former model included a history of diabetes as a risk variable and identified it as an independent risk factor for SMI (OR = 2.30, 95% CI: 1.20–4.43). However, diabetes history can only reflect the clinical background, not the dynamic changes in blood glucose. Abnormal glucose metabolism is the most prominent clinical feature of diabetes mellitus, and insulin resistance is main pathological basis. In this study, the patient’s glucose, lipid and obesity were combined to TyG-BMI. The TyG-BMI was used as a study variable to better reflect the metabolic level of patients, and the results showed high predictive accuracy.

The use of a predictive model in clinical practice and improvements in decision making are discussed below. (1) The model is used for routine health screening of the general population. Through comprehensive analysis of general population information and routine blood biochemical indicators such as fasting blood glucose and blood lipids, it can achieve early identification of risk factors related to the development of SMI and dietary or pharmacological interventions to reduce its occurrence of SMI. (2) It helps physicians to identify high-risk patients and guide patients to further improve coronary angiography or coronary angiography.

Limitations of this study include, (1) The study excluded patients with prior myocardial infarction, potentially limiting the model’s ability to assess recurrent SMI risk in this population. (2) The OR values for TyG-BMI and UA showed narrow confidence intervals. There may be some correlation between these variables, which limited the direct correlation between these variables and SMI. The reasons for this correlation may include: First, obesity and insulin resistance are strongly associated with UA levels. Obesity promotes UA production and decreases excretion. Then, TyG-BMI exacerbates metabolic inflammation and disrupts UA homeostasis [34]. (3) Despite our adjustment for covariates, there were residual confounding variables (lifestyle factors, family history, psychological status) that may have weakened this direct relationship. (4) The small sample size and data from a single center are not easily generalizable. (5) Only internal verification of the model has been performed, no external verification of the model has been performed.

Future research directions include, (1) Conducting multi-centre studies, increasing sample size and including more covariates. (2) Analysing the mediating effects of all variables and exploring the causal relationship between variables. (3) Conduct subgroup analysis to test the independence of variables.

## 5. Conclusions 

This study shows that six factors, including gender, age, HDL-C, ApoB/A1, UA, and TyG-BMI, are closely associated with the occurrence of SMI in MI patients. The model developed demonstrated excellent performance in discrimination, predictive consistency, and clinical applicability. By incorporating emerging indicators such as the TyG-BMI index, the model improves the prediction of SMI risk and provides clinicians with a more comprehensive assessment tool.

## Availability of Data and Materials

The raw data supporting the conclusions of this article will be made available by the authors, without undue reservation.
